# 2017. Comparison of California Acute Care Hospital Central Line-Associated Bloodstream Infection Incidence by Hospital Location Before and During the COVID-19 Pandemic

**DOI:** 10.1093/ofid/ofac492.1641

**Published:** 2022-12-15

**Authors:** Lizette Brenes, Monise Magro, Tisha Mitsunaga, Erin Epson

**Affiliations:** California Department of Public Health (CDPH), Richmond, California; California Department of Public Health, Richmond, California; California Department of Public Health, Richmond, California; California Department of Public Health, Richmond, California

## Abstract

**Background:**

Central line-associated bloodstream infection (CLABSI) incidence among acute care hospitals (ACH) and in some patient care locations such as critical care (CC) units increased substantially nationwide during the COVID-19 pandemic. We compared California ACH CLABSI incidence by location before and during the pandemic to identify locations with high burden to inform targeted prevention efforts.

**Methods:**

Using California ACH (n=327) CLABSI standardized infection ratio (SIR) data from the National Healthcare Safety Network, we compared incidence during the second halves of 2019 (2019H2) and 2020 (2020H2) to evaluate early pandemic changes, and during 2019 (pre-pandemic) and 2021 (pandemic) periods by hospital type, location type (e.g., CC), and patient care location (e.g., medical CC), excluding rehabilitation units. A mid-P exact test was applied to compare SIRs between study periods.

**Results:**

ACH CLABSI SIR increased statewide from 2019H2 to 2020H2 by 50.8% (0.65 to 0.98) and from 2019 to 2021 by 34.3% (0.67 to 0.90). Community hospitals < 125 beds had the highest SIR and percentage increase in 2021 as well as 2020H2. Of 9 location types and 58 patient care locations, CC units and medical and medical-surgical CC had significantly higher SIR in both comparisons (2020H2 versus 2019H2 and 2021 versus 2019); wards and medical wards in 2020H2 only, and step-down units and adult step-down in 2021 only. Trauma CC SIR was significantly higher only in 2020H2 compared to 2019H2, while surgical CC SIR was significantly higher in 2021 compared to 2019. Respiratory CC had the highest SIR (2.99, 95%CI 2.14-4.08) in 2021, but was not significantly higher when compared to 2019 (1.06, 95%CI 0.21-3.41).

**Figure:**
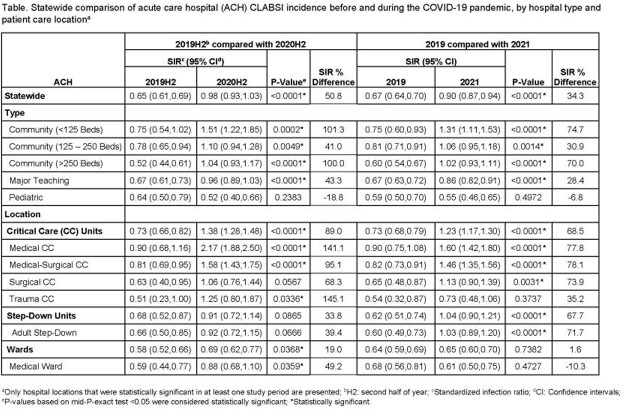

**Conclusion:**

We observed an overall statewide increase in hospital CLABSI incidence, especially in CC locations, during the pandemic. Although the SIR increase relative to pre-pandemic was smaller in 2021 than in 2020H2, with some exceptions, most locations had persistently higher incidence. We will further assess associations between CLABSI incidence and antimicrobial resistance, device insertion dates, and hospital COVID-19 burden. We will use our findings to guide public health support for hospital infection prevention programs to reduce their CLABSI incidence.

**Disclosures:**

**All Authors**: No reported disclosures.

